# Supplementary material for: Role of Dorsomedial Hypothalamus GABAergic Neurons in Sleep–Wake States in Response to Changes in Ambient Temperature in Mice
Source: Int J Mol Sci. 2022 Jan 23;23(3):1270. doi: 10.3390/ijms23031270 (PMC8836016; doi:10.3390/ijms23031270)
Supplement: Supplementary file 1 [file ijms-23-01270-s001.zip › ijms-1522563-supplementary.pdf]

Supplementary materials

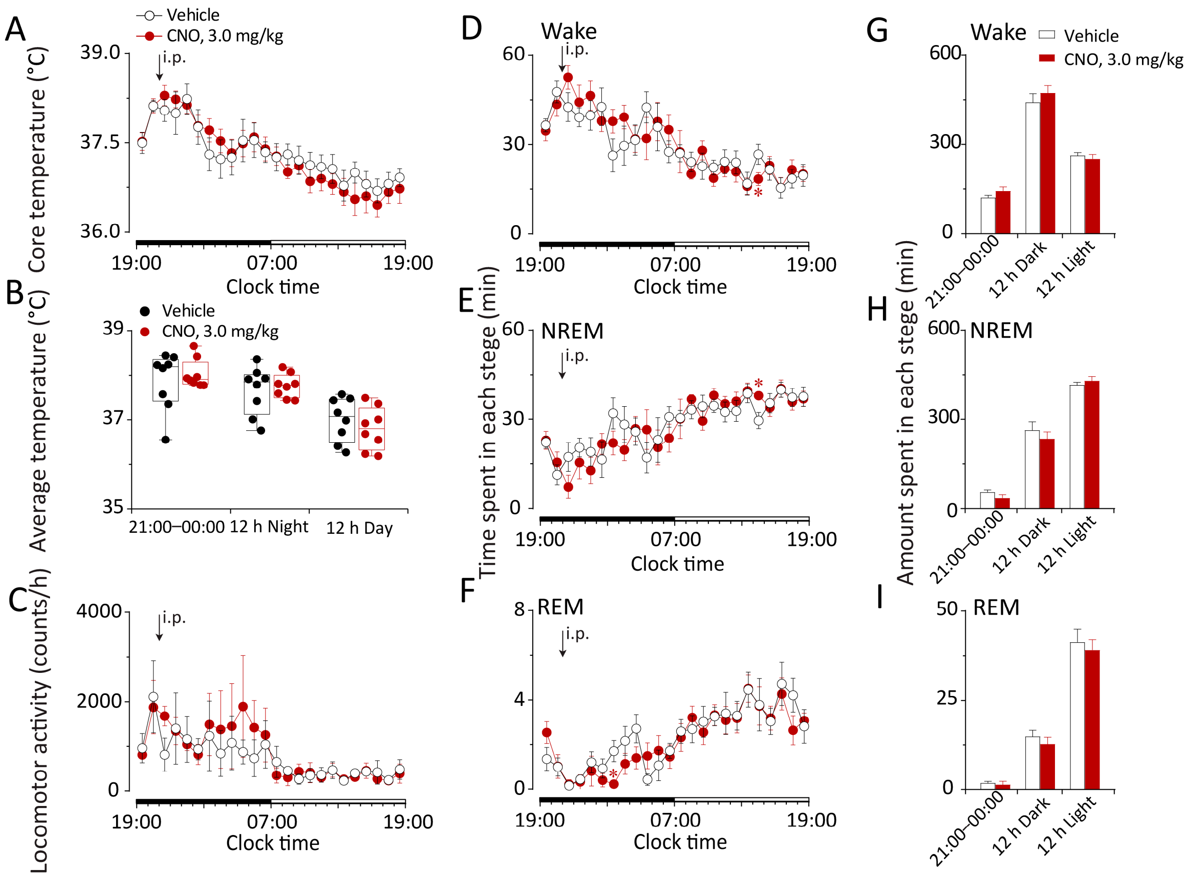

Figure S1. Chemogenetic activation of DMH GABAergic neurons did not affect the core temperature or sleep–wake cycle in vGAT–Cre mice.

(**A**) Time–course of the core body temperature of vGAT–Cre mice expressing hM3Dq in the DMH after the administration of vehicle or CNO (3 mg/kg) at 21:00. (**B**) Average values of the core body temperature during 4h after the administration of vehicle or CNO (3 mg/kg) at 21:00. (**C**) Time–course of the locomotion after the administration of vehicle or CNO (3 mg/kg) at 21:00. (**D–F**) Time courses of each stage of vGAT–Cre mice expressing hM3Dq in the DMH after the administration of vehicle or CNO (3 mg/kg) at 21:00. (**G–I**) Amounts of each stage during 3 h, 12 h Light or 12 h Dark after the administration of vehicle or CNO (3 mg/kg). Values were presented as means ± S.E.M (n = 7–8). * p < 0.05 indicated significant differences from the vehicle group as assessed by repeated ANOVA (D and F).

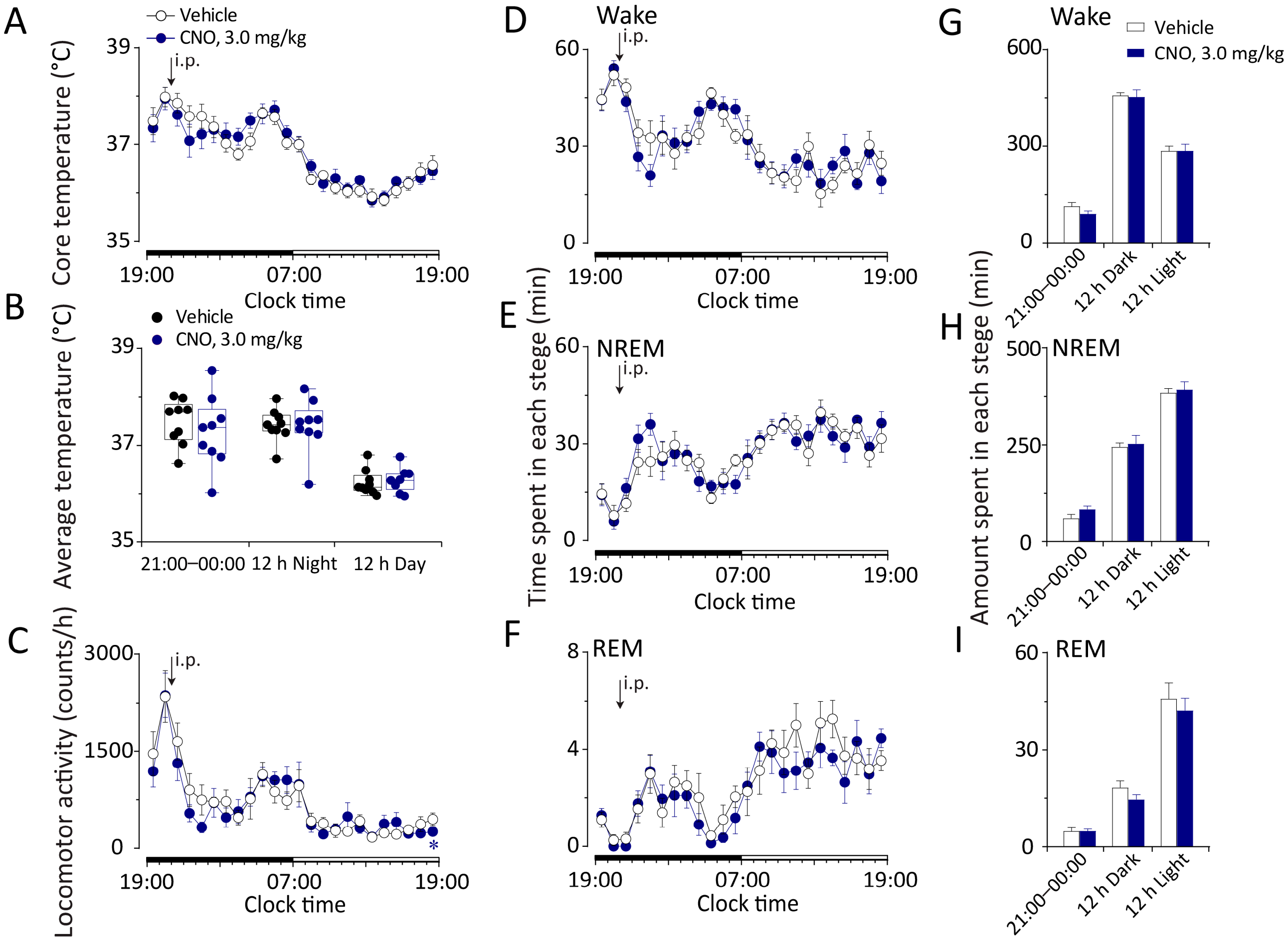

Figure S2. Chemogenetic inhibition of DMH GABAergic neurons did not affect sleep–wake cycle in vGAT–Cre mice.

(**A**) Time–course of the core body temperature of vGAT–Cre mice expressing hM4Di in the DMH after the administration of vehicle or CNO (3 mg/kg) at 21:00. (**B**) Average values of the core body temperature during 4h after the administration of vehicle or CNO (3 mg/kg) at 21:00. (**C**) Time courses of the locomotion after the administration of vehicle of CNO (3 mg/kg) at 21:00. (**D–F**) Time courses of each stage of vGAT–Cre mice expressing hM4Di in the DMH after the administration of vehicle or CNO (3 mg/kg) at 21:00. (**G–I**) Amounts of each stage during 3 h, 12 h daytime or 12 h nighttime after the administration of vehicle or CNO (3 mg/kg). Values were presented as means ± S.E.M (n = 7–8).

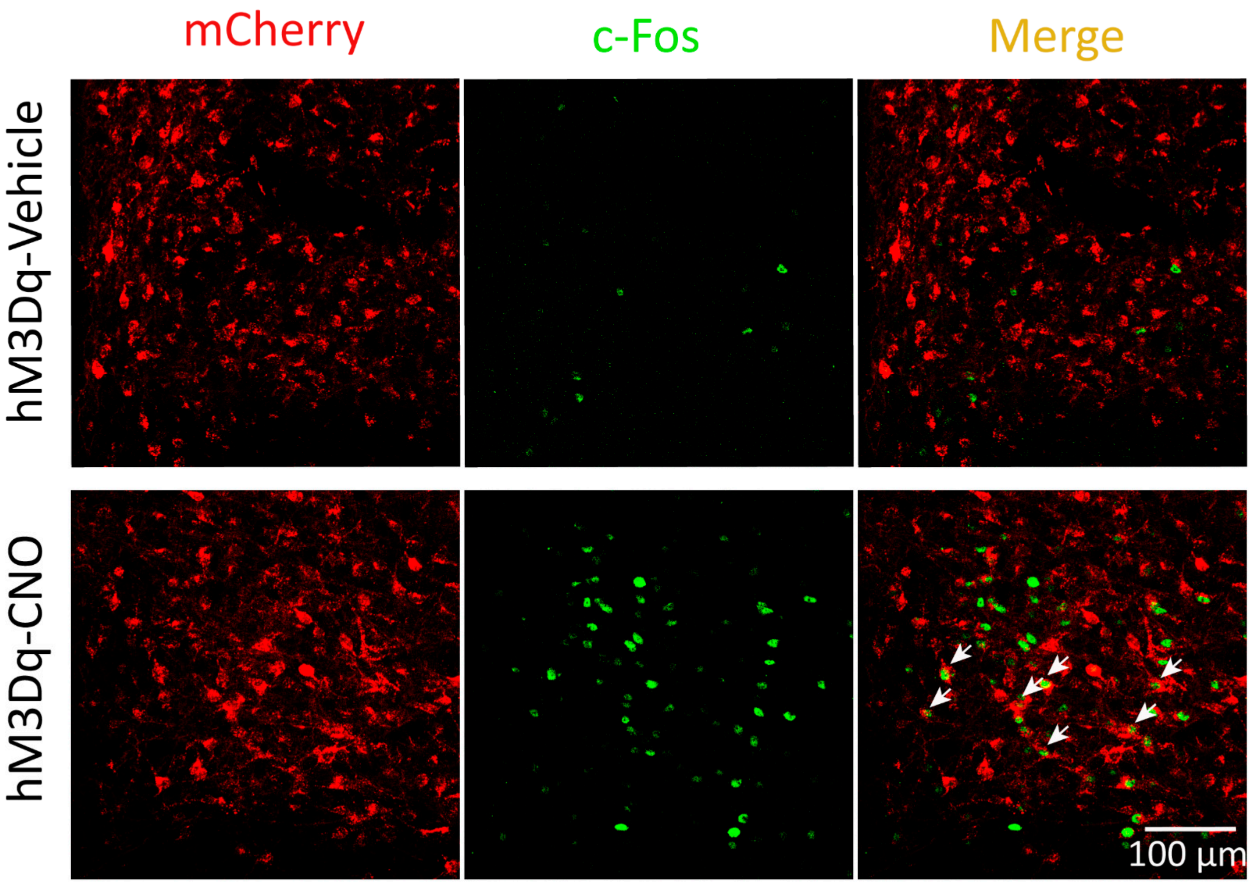

Figure S3. CNO induced c–Fos experssion compared with vehicle group in hM3Dq–vGAT–Cre mice.

Left panel: The mCherry expression representing the hM3Dq expression (red) in the DMH. Middle panel: The c–Fos expression (green) in the DMH. Right panel: The merge image (yellow) of mCherry and c–Fos in the DMH. White arrows indicated the double-stained of mCherry and c–Fos. Scale bar: 100 μm.
