# Supplementary figures and images for: Role of Dorsomedial Hypothalamus GABAergic Neurons in Sleep–Wake States in Response to Changes in Ambient Temperature in Mice
Source: Int J Mol Sci. 2022 Jan 23;23(3):1270. doi: 10.3390/ijms23031270 (PMC8836016; doi:10.3390/ijms23031270)

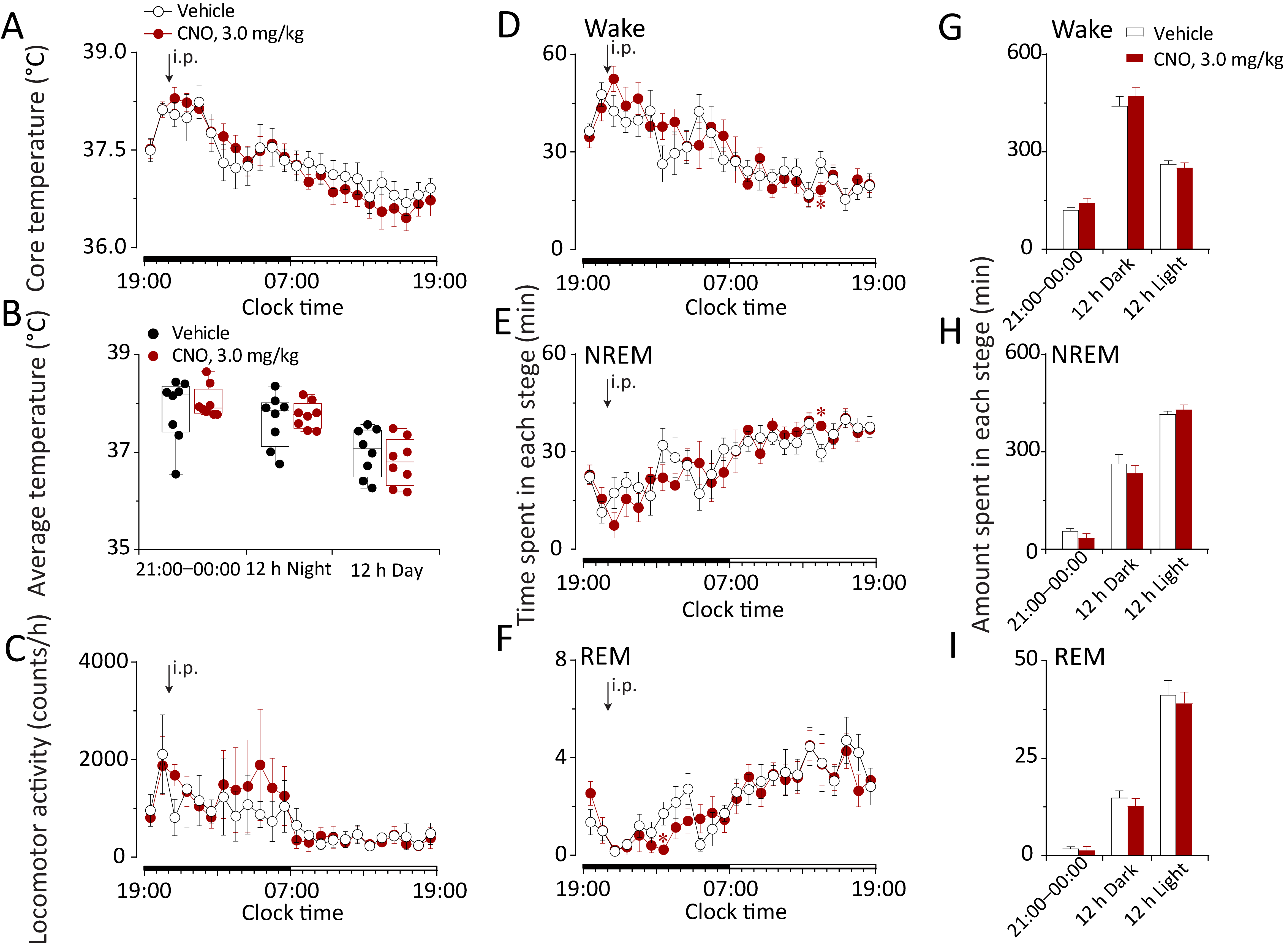

Supplement: Supplementary file 1 [file ijms-23-01270-s001.zip › Figure S1.tif]

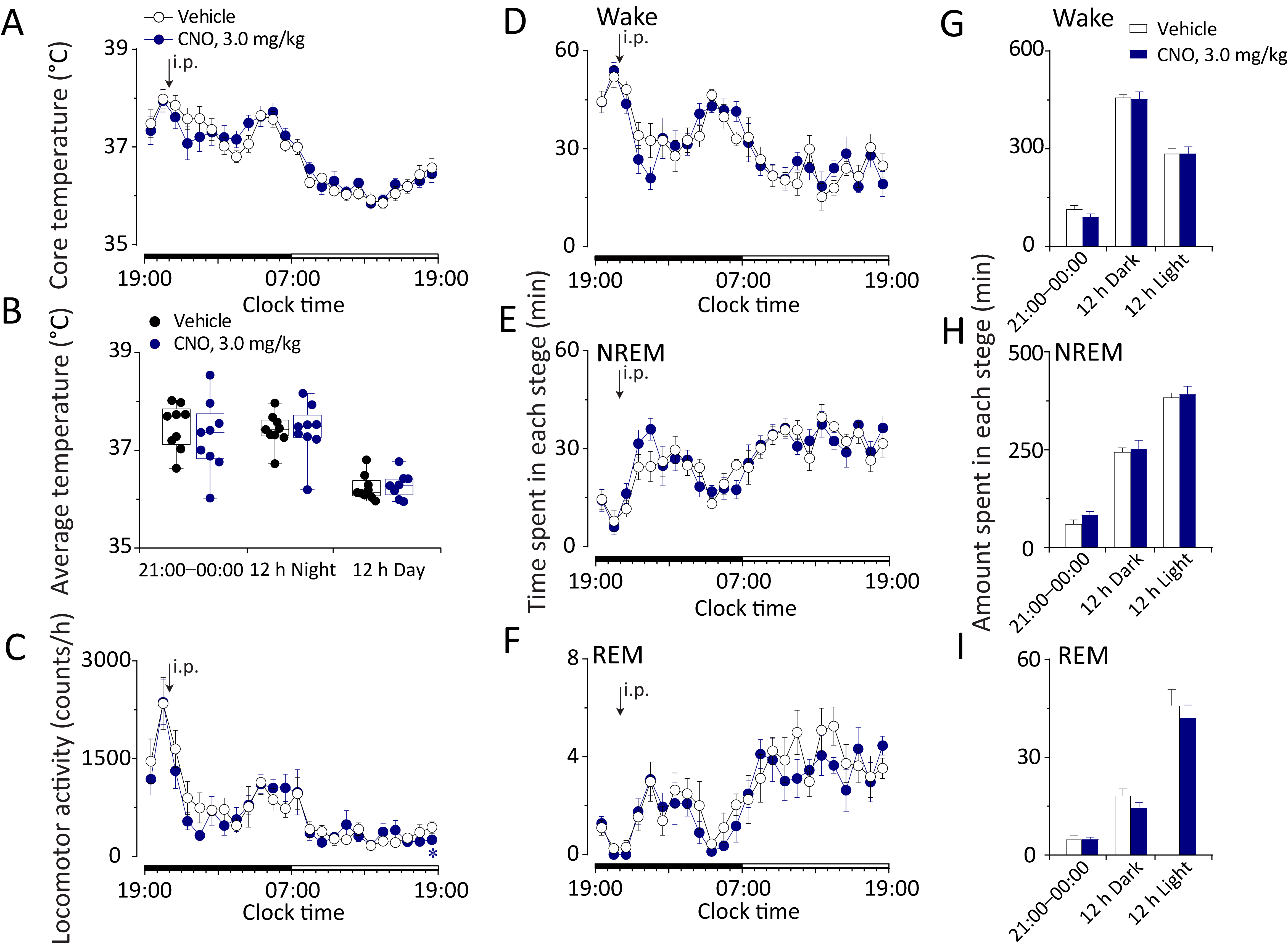

Supplement: Supplementary file 1 [file ijms-23-01270-s001.zip › Figure S2.tif]

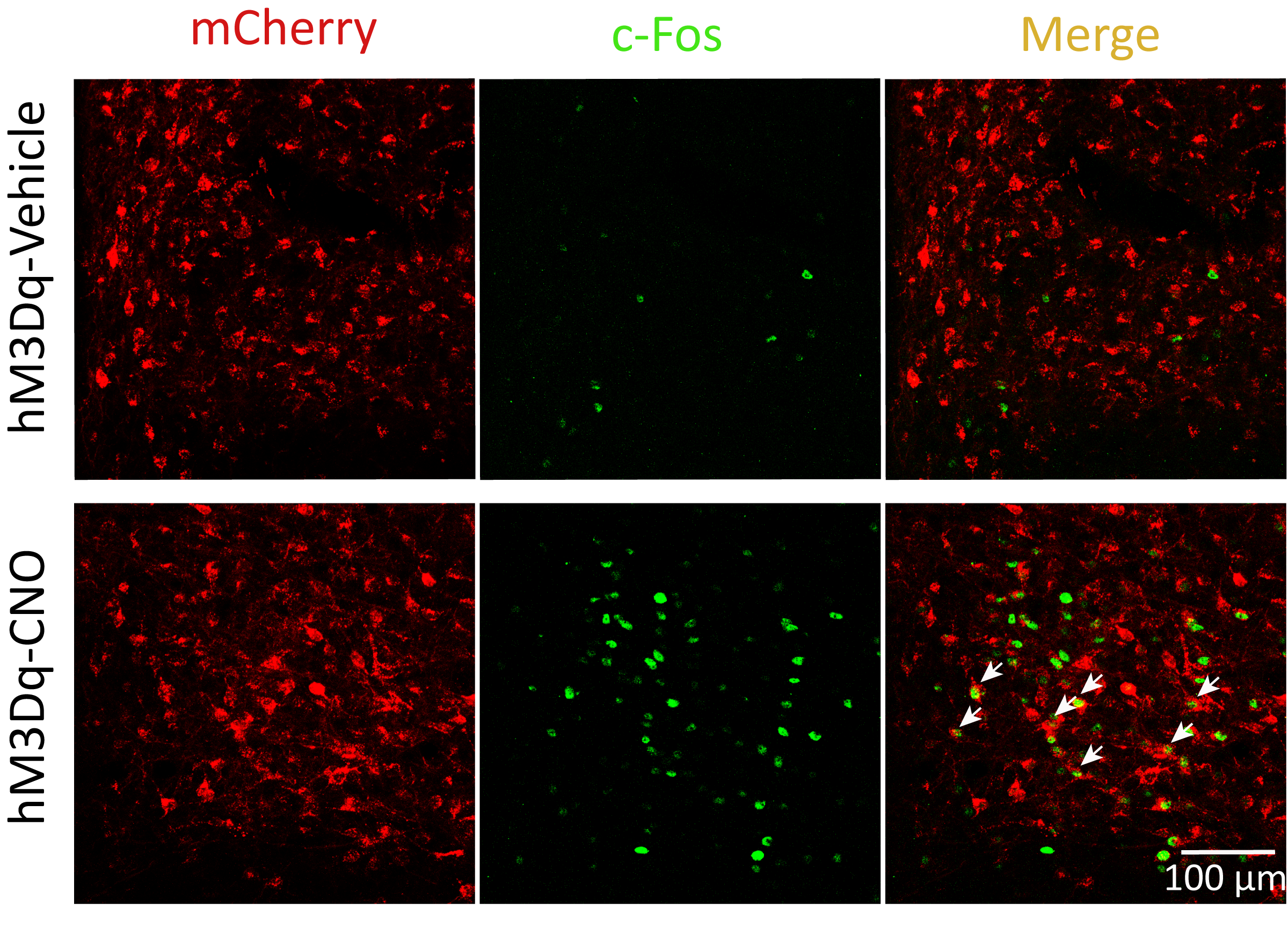

Supplement: Supplementary file 1 [file ijms-23-01270-s001.zip › Figure S3.tif]
